# Impact of vitamin D on immune function: lessons learned from genome-wide analysis

**DOI:** 10.3389/fphys.2014.00151

**Published:** 2014-04-21

**Authors:** Rene F. Chun, Philip T. Liu, Robert L. Modlin, John S. Adams, Martin Hewison

**Affiliations:** ^1^Department of Orthopaedic Surgery, Orthopedic Hospital Research Center, David Geffen School of Medicine, University of California at Los AngelesLos Angeles, CA, USA; ^2^Division of Dermatology, Department of Medicine, David Geffen School of Medicine, University of California at Los AngelesLos Angeles, CA, USA

**Keywords:** macrophage, dendritic cell, intracrine, antigen-presentation, antibacterial, CYP27B1, VDR

## Abstract

Immunomodulatory responses to the active form of vitamin D (1,25-dihydroxyvitamin D, 1,25D) have been recognized for many years, but it is only in the last 5 years that the potential role of this in normal human immune function has been recognized. Genome-wide analyses have played a pivotal role in redefining our perspective on vitamin D and immunity. The description of increased vitamin D receptor (VDR) and 1α-hydroxylase (CYP27B1) expression in macrophages following a pathogen challenge, has underlined the importance of intracrine vitamin D as key mediator of innate immune function. It is now clear that both macrophages and dendritic cells (DCs) are able to respond to 25-hydroxyvitamin D (25D), the major circulating vitamin D metabolite, thereby providing a link between the function of these cells and the variations in vitamin D status common to many humans. The identification of hundreds of primary 1,25D target genes in immune cells has also provided new insight into the role of vitamin D in the adaptive immune system, such as the modulation of antigen-presentation and T cells proliferation and phenotype, with the over-arching effects being to suppress inflammation and promote immune tolerance. In macrophages 1,25D promotes antimicrobial responses through the induction of antibacterial proteins, and stimulation of autophagy and autophagosome activity. In this way variations in 25D levels have the potential to influence both innate and adaptive immune responses. More recent genome-wide analyses have highlighted how cytokine signaling pathways can influence the intracrine vitamin D system and either enhance or abrogate responses to 25D. The current review will discuss the impact of intracrine vitamin D metabolism on both innate and adaptive immunity, whilst introducing the concept of disease-specific corruption of vitamin D metabolism and how this may alter the requirements for vitamin D in maintaining a healthy immune system in humans.

## Introduction

Amongst the many reported extra-skeletal effects of vitamin D, its ability to regulate immunity through effects on both the innate and adaptive systems has received considerable attention. This stems in part from homage to studies carried out more than a century ago by a then relatively unknown scientist, Dr. Nils Finsen. In 1903 Dr. Finsen won the Nobel Prize for Medicine or Physiology for showing that he could cure the epidermal form of tuberculosis (TB), lupus vulgaris, using concentrated light irradiation (Moller et al., 2005). The subsequent discovery that exposure to ultra-violet light promotes epidermal synthesis of vitamin D led to further studies describing the successful use of oral vitamin D supplementation to treat lupus vulgaris, and other mycobacterial infections such as leprosy (Airey, 1946; Herrera, 1949). The advent of antibiotic therapies for infectious diseases appeared to have consigned these studies to the history books. However, in 2006 the work of Finsen returned to center stage as a consequence of a series of genome-wide analyses that revealed pathogen-induction of an intracrine vitamin D system in monocytes (Liu et al., 2006), and an associated mechanism for anti-mycobacterial actions of vitamin D (Wang et al., 2004), whilst also shedding light on how these responses may vary according to the vitamin D “status” of any given individual. With increasing awareness of vitamin D-deficiency across the globe (Holick, 2007), and ongoing discussions concerning the physiological and clinical relevance of this (Holick et al., 2011; Ross et al., 2011), these genome-wide analyses have played a pivotal role in defining our new perspective on non-classical vitamin D physiology. The current review will detail these developments and how they have helped to define a role for vitamin D in normal immune function.

## Antibacterial responses to vitamin D

Despite its early use in the treatment of mycobacterial diseases such as TB and leprosy (Airey, 1946; Herrera, 1949), the immunomodulatory actions of vitamin D did not become clear until much later. Elucidation of this important non-classical action of vitamin D stemmed from two key observations. Firstly, most proliferating cells within the immune system express the nuclear receptor for active 1,25-dihydroxyvitamin D (1,25D)—the vitamin D receptor (VDR). Initial studies focused on 1,25D binding capacity in cells from the adaptive immune system such as T and B lymphocytes (T and B cells) (Bhalla et al., 1983; Provvedini et al., 1983), with subsequent reports describing specific intracellular binding of 1,25D in cells from the innate immune system such as monocytes/macrophages (Kreutz et al., 1993), dendritic cells (DC) (Brennan et al., 1987), neutrophils (Takahashi et al., 2002), and monocytic cell lines (Mangelsdorf et al., 1984). The functional significance of these data was not immediately clear but, nevertheless, it was assumed that VDR-expressing immune cells were able to respond the circulating active 1,25D in a similar fashion to classical vitamin D target tissues such as the intestine, kidney, and bone. However, this assumption was challenged by the second major observation linking vitamin D and the immunity, namely the discovery of active vitamin D metabolism by cells from the immune system.

Elevated serum levels of 1,25D reported for some patients with the granulomatous disease sarcoidosis were shown to be due to conversion of pro-hormone 25D to 1,25D by tissue and systemic macrophages in these patients (Barbour et al., 1981; Adams et al., 1983). Similar observations for other inflammatory and granulomatous diseases (Kallas et al., 2010) suggested that immune activity of the enzyme that catalyzes metabolism of 25D to 1,25D, 25-hydroxyvitamin D-1α-hydroxylase (1α-hydroxylase) was a disease-related phenomenon. However, other studies, *in vitro*, highlighting the potential for macrophage 1α-hydroxylase activity in the absence of disease (Koeffler et al., 1985; Reichel et al., 1986) supported the exciting possibility that synthesis of 1,25D is part of normal immune function. Despite this, it was another 20 years before evidence to support this proposal was reported. Significantly, the major advances that provided this evidence involved genome-wide strategies that explored both the regulation and function of vitamin D by immune cells.

**Figure 1:**
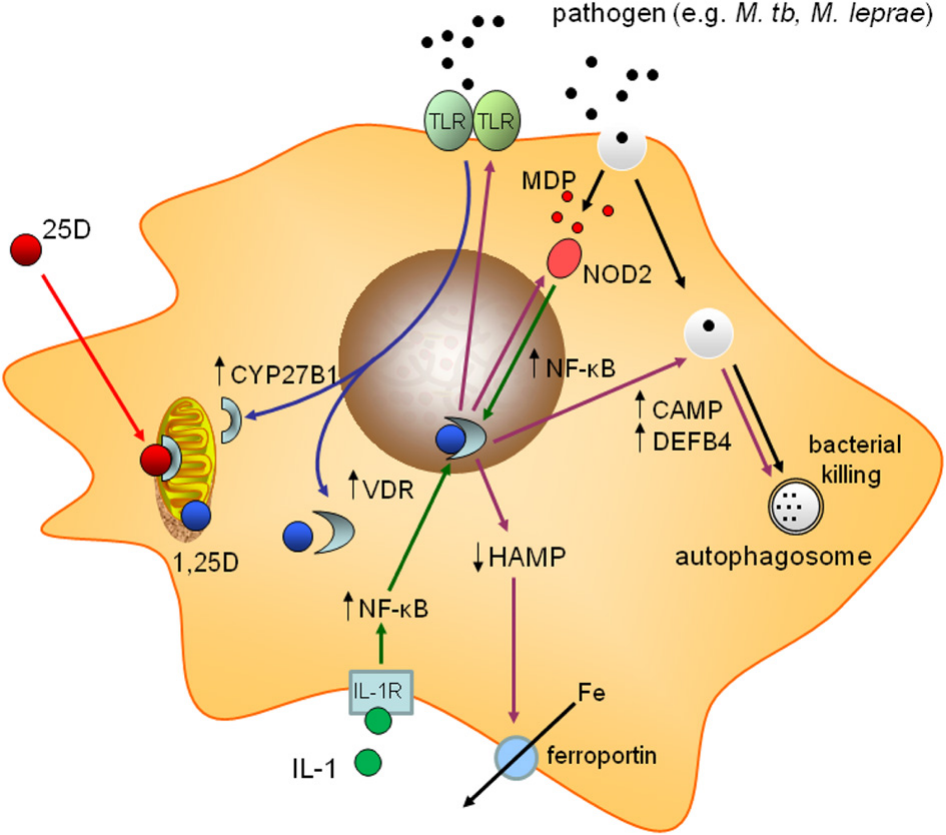
**Mechanisms for induction of vitamin D-mediated antibacterial responses in monocytes.** Schematic representation of monocyte/macrophage responses to infection with a pathogen such as *Mycobacterium tuberculosis* (*M. tb*). Pattern recognition receptors (TLR2/1) sense *M. tb* and signal to induce expression of 1α-hydroxylase (CYP27B1) and the vitamin D receptor (VDR). The resulting intracrine system for vitamin D (blue arrows) converts 25-hydroxyvitamin D (25D) to 1,25-dihydroxyvitamin D (1,25D), which then binds to VDR and promotes transcriptional regulation. Prominent responses to intracrine activation of vitamin D (pink arrows) include: induction of antibacterial cathelicidin (CAMP) and β-defensin 2 (DEFB4); suppression of iron-regulatory hepcidin (HAMP); promotion of autophagy; induction of NOD2 expression; feedback regulation of toll-like receptor (TLR) expression; increased bacterial killing. For some responses (e.g., induction of DEFB4) accessory immune signals (MDP binding to NOD2, and IL-1 responsiveness) cooperate with intracrine vitamin D via nuclear factor-κB (NF-κB) (green arrows).

The first of the genome-wide studies to shed light on extra-skeletal actions of vitamin D was published by John White and colleagues at McGill University in Montreal and utilized a combination of DNA array and *in silico* strategies. In this report, DNA array analysis of 1,25D-regulated genes in squamous cell carcinoma cells *in vitro* (Akutsu et al., 2001; Lin et al., 2002) was combined with *in silico* analysis of genomic VDR binding sites to provide a comprehensive overview of potential 1,25D-VDR target genes (Wang et al., 2005). Genome-wide analysis of DNA sequences that are able to bind liganded VDR revealed consensus vitamin D response elements (VDRE) within the gene promoters for two antibacterial proteins, cathelcidin (*CAMP*) and β-defensin 2 (*DEFB4*) (Wang et al., 2005). Interestingly, although both of these genes exhibited classical proximal promoter direct-repeat 3 (DR3) consensus VDREs, only *CAMP* appeared to be transcriptionally induced by 1,25D in monocytes (Wang et al., 2005). The underlying mechanism for the differential regulation of monocyte *CAMP* and *DEFB4* by 1,25D was elucidated in subsequent studies, the first of which described increased expression of monocyte *DEFB4* following co-treatment with 1,25D and the inflammatory cytokine interleukin-1 (IL-1) (Liu et al., 2009). Based on these observations and promoter analysis for the *CAMP* and *DEFB4* genes, it was concluded that transcriptional induction of *DEFB4* requires cooperative occupancy of nuclear factor-κB (NF-κB) response elements as well as VDRE within the *DEFB4* gene promoter. By contrast, induction of *CAMP* appears to be primarily dependent on binding of VDR to promoter VDRE (Liu et al., 2009). The importance of NF-κB and VDR as co-inducers of *DEFB4* transcription was further emphasized by studies of the intracellular pathogen sensing protein NOD2 which is itself transcriptionally induced by 1,25D (Wang et al., 2010b). Cells co-treated with 1,25D and the ligand for NOD2, muramyl dipeptide (MDP), a cell wall product of Gram-positive and Gram-negative bacteria, showed potent NF-κB-dependent induction of *DEFB4* (Wang et al., 2010b). In these studies expression of *CAMP* was also enhanced by 1,25D-MDP co-treatment, suggesting that NF-κB may cooperate with VDR in a variety of immunomodulatory functions (Figure 1).

Subsequent functional studies confirmed that dose-dependent vitamin D induction of *CAMP* transcription involves direct interaction between liganded VDR and VDRE in the *CAMP* gene promoter (Wang et al., 2004; Gombart et al., 2005). Intriguingly, the VDRE initially identified within the *CAMP* promoter appear to be specific for subhuman and human primates, as there are no similar motifs within equivalent genes for lower mammals (Gombart et al., 2005). Acquisition of a VDRE by the *CAMP* gene appears to have occurred following the introduction of an Alu short interspersed nuclear element (SINE) that placed *CAMP* under the control of 1,25D-VDR (Gombart et al., 2009b). This primate-specific adaptation has been conserved in humans and apes as well as Old World and New World primates, suggesting that a mechanism for transcriptional regulation of *CAMP* by vitamin D confers biological advantages. The assumption is that this mechanism will be potently activated by the relatively high circulating levels of 25D and 1,25D that are characteristic of non-human primates (Adams et al., 1985). A similar mechanism would also have been advantageous in early *Homo sapiens* whose existence is likely to have been defined by routine exposure to ultra-violet light and increased cutaneous vitamin D production, with associated high circulating levels of 25D. Conversely, in modern man where serum 25D status is more variable, this antibacterial mechanism may be less effective. Whilst this hypothetical mechanism broadly supports beneficial innate immune effects of vitamin D supplementation, the important question still remaining is how variations in serum levels of inactive 25D are able to influence immune responses driven by intracellular 1,25D and VDR. The answer to this question has been pivotal to our perspective on the non-classical actions of vitamin D and was, again, provided by genome-wide analyses.

For cells from both within and outside the classical immune system, recognition of and response to pathogens involves surveillance of pathogen-associated molecular patterns (PAMPs) by pattern recognition receptors (PRR). Prominent amongst these PRRs is the extended family of Toll-like receptor (TLR) non-catalytic transmembrane receptors which interact with specific PAMPs (Moresco et al., 2011). To clarify the role of the TLR2/1 heterodimer in mediating innate immune responses to the TB pathogen, *Mycobacterium tuberculosis* (*M. tb*), Liu and colleagues carried out DNA array analysis of gene expression in human macrophages and DCs following treatment with one of the putative PRRs for *M. tb* (Liu et al., 2006). Amongst the many macrophage genes shown to be differentially regulated by 19 kDa lipoprotein (a TLR2-interacting PAMP), expression of both *CYP27B1* and *VDR* was increased (Liu et al., 2006) (see Figure 1). This genome-wide approach provided the first unbiased evidence of a role for vitamin D metabolism and signaling in innate immune responses to a pathogen. Crucially, the TLR2/1-stimulus induced expression of both *CYP27B1* and *VDR*, suggesting that macrophage responses to *M. tb* involve an endogenous, intracrine, vitamin D system. Further studies showed that macrophages co-treated with the TLR2/1-ligand, 19 kDa lipoprotein were responsive to both active 1,25D and inactive 25D, confirming the functional efficacy of the intracrine model. Moreover, inhibition of either 1α-hydroxylase activity, or VDR function blocked the actions of 25D, underlining the importance of cell-specific metabolism as a determinant of vitamin D immunoregulation.

The specific functional read-outs used in the TLR2/1-*M.tb* study were induction of mRNA for the vitamin D catabolic enzyme 24-hydroxylase (*CYP24A1)* and the antibacterial protein *CAMP* (Liu et al., 2006). Parallel analysis of the other antibacterial target gene for 1,25D, *DEFB4*, did not reveal significant induction of this gene in the presence of 19 kDa lipoprotein and 25D. However, as outlined above, subsequent experiments demonstrated that co-treatment with either IL-1 (Liu et al., 2009), or the NOD2 ligand MDP (Wang et al., 2010b) cooperates with the TLR2/1 ligand and 25D to stimulate expression of *DEFB4*. Antibacterial proteins such as CAMP and DEFB4 play a crucial role in vitamin D-mediated killing of intracellular bacteria. Monocytes treated with increasing concentrations of CAMP peptide show a dose-dependent decrease in the viability of internalized *M. tb* (Liu et al., 2006); a similar inhibition of macrophage *M. tb* viability occurs in the presence of 25D, with this effect being abrogated by a VDR antagonist. In other studies knockdown of either *CAMP* or *DEFB4* decreased killing of *M. tb* in macrophages, suggesting that both antibacterial proteins are important in mediating vitamin D-induced responses to mycobacterial infection (Liu et al., 2009). Both CAMP and DEFB4 are detectable in the circulation where they are able to support innate immune responses to extra-cellular pathogens including anti-viral responses (Barlow et al., 2011; Tripathi et al., 2013). However, pathogens such as mycobacteria are internalized by phagocytosis, and bacterial killing can then take place following fusion of the resulting phagosome with a lysosome to form a phagolysosome. To evade this antibacterial process and maintain intracellular viability *M. tb* can subvert the transition of phagosomes to phagolysosomes (Vergne et al., 2004). In this situation, the host cell can restore pathogen encapsulation by evoking alternative mechanisms such as autophagy, in which encapsulation of organelles, cell proteins or intracellular pathogens in a double-membrane autophagosome occurs prior to fusion with lysosomes (Gutierrez et al., 2004). Although a well-recognized feature of eukaryotic cells cytosolic homeostasis (Klionsky and Emr, 2000), autophagy also appears to play a pivotal role in cellular response to infection (Gutierrez et al., 2004; Deretic and Levine, 2009). The ability of 1,25D and its synthetic analogs to promote autophagy is well established (Hoyer-Hansen et al., 2005; Wang et al., 2008), but recent data suggest that induction of autophagy may be particularly important for vitamin D-induced antibacterial responses to *M. tb* infection (Yuk et al., 2009; Shin et al., 2011) (Figure 1). The precise mechanism for this is not clear and may involve inhibition of the mammalian target of rapamycin (mTOR) intracellular signaling system (O'Kelly et al., 2006; Lisse et al., 2011) with mTOR acting to suppress the induction of autophagy (Sanjuan et al., 2009). It has also been suggested that vitamin D-induced autophagy occurs via an indirect mechanism, in that RNA-interference (RNAi) knockdown of antibacterial *CAMP* was sufficient to abrogate 1,25D-induced autophagy in monocytes (Yuk et al., 2009). In common with effects on expression of antibacterial proteins, it was noted that monocyte autophagy following activation of TLR2/1 involves enhanced expression of VDR and CYP27B1 (Shin et al., 2011), further highlighting the importance of intracrine 25D metabolism and action in normal human innate immunity.

Intracrine synthesis of 1,25D has also been shown to regulate expression of another antibacterial protein, hepcidin antibacterial protein (HAMP) (Bacchetta et al., 2013b). However, in contrast to CAMP and DEFB4, the direct microbiocidal properties of HAMP appear to be relatively weak. Instead, the major function of HAMP appears to be suppression of the cell membrane protein ferroportin, the only known exporter of intracellular iron (Ganz, 2011). This link between HAMP and ferroportin in cells such as enterocytes, hepatocytes and monocytes plays a key role in the so-called anemia of infection or chronic disease (Ganz, 2009). Because pathogens such as bacteria utilize iron to maintain growth, restriction of circulating iron concentrations provides an important host response to systemic infection (Drakesmith and Prentice, 2012). However, for pathogens such as *M. tb* that attempt to evade immune surveillance at the intracellular level, accumulation of iron within this environment may promote the growth of internalized pathogens such as *Salmonella typhimurium* (Nairz et al., 2007), *M. tb* (Schaible et al., 2002; Sow et al., 2007, 2009), and *Chlamydia psittaci* (Paradkar et al., 2008). Conversely, innate immune and viral stimuli are known to stimulate the expression of *HAMP* (Sow et al., 2009; Armitage et al., 2011). In this setting suppression, rather than induction, of HAMP by 25D and 1,25D may be beneficial by abrogating HAMP-induced suppression of ferroportin which, in turn, will favor iron export and lower intracellular concentrations of iron. In studies carried out by our group at UCLA, we have shown that 25D and 1,25D suppress transcription of *HAMP* in monocytes and hepatocytes, leading to increased membrane expression of ferroportin, and decreased expression of ferritin (a surrogate biomarker for intracellular iron concentrations) (Bacchetta et al., 2013b). Moreover, in contrast to CAMP and DEFB4, elevated serum 25D levels (but not 1,25D) following vitamin D supplementation of human subjects *in vivo* were associated with potent suppression of circulating concentrations of HAMP (Bacchetta et al., 2013b). It therefore appears that regulation of the HAMP-ferroportin axis is another key facet of vitamin D-mediated innate immune function, complimentary to its reported effects on antibacterial proteins (Liu et al., 2006; Adams and Hewison, 2008; Hewison, 2011), and autophagy (Yuk et al., 2009; Shin et al., 2011) (see Figure 1). However, it is important to recognize that the effect of vitamin D on serum levels of hepcidin may have additional consequences that are both positive (suppression of anemia) and negative (decreased hepcidin for systemic infection). This may be particularly important for patients with chronic kidney disease (CKD) who commonly present with impaired circulating levels of 25D and 1,25D, and who are at higher risk of infection. In CKD, low serum 25D has been shown to correlate inversely with anemia (Lac et al., 2010) and directly with blood hemoglobin levels (Kiss et al., 2011). These effects may be due to dysregulation of normal HAMP-ferroportin function under conditions of vitamin D-deficiency, further emphasizing the importance of vitamin D supplementation in these patients.

Vitamin D may also target other innate immunity mechanisms. For example, studies *in vitro* have shown that 1,25D promotes hyporesponsiveness to PAMPs by downregulating expression of TLR2 and TLR4 on monocytes (Sadeghi et al., 2006). In this way, vitamin D appears to promote feedback control pathways that limit antibacterial activity and other innate immune responses, thereby preventing potential inflammatory events that arise from an over-elaboration of immune responses, notably inflammatory T cell responses. Paradoxically, vitamin D can also promote responses that amplify innate immune function. Recent studies have described 1,25D-mediated induction of the triggering receptor on myeloid cells-1 (TREM-1) (Kim et al., 2013), a cell surface protein associated with cytokine and chemokine production (Bouchon et al., 2000) that can also act to amplify TLR signaling (Bouchon et al., 2001). The biological significance of this is still not clear and this mechanism may be more important for cells such as neutrophils which are the principal source of circulating CAMP. Neutrophils express VDR but, unlike monocytes/macrophages, they do not appear to express a functional 1α-hydroxylase and are therefore not subject to intracrine activation of innate immune function. In this setting, activation of proteins such as TREM-1 may help to promote neutrophil responses to circulating 1,25D rather than 25D through enhanced TLR-signaling. This, in turn, would stimulate expression of VDR and sensitivity to 1,25D.

A similar cooperative TLR response has also been described for epithelial keratinocytes, where basal expression of *CYP27B1* is insufficient to facilitate intracrine induction of antibacterial proteins by serum 25D. However, following skin wounding, locally generated transforming growth factor β (TGFβ) enhances expression of CYP27B1 (Schauber et al., 2007). The resulting TGFβ-driven *CYP27B1* expression is then able to stimulate intracrine generation of antibacterial proteins such as CAMP to combat potential infections associated with epidermal injury (Schauber et al., 2007). Interestingly, the TGFβ-induced 1α-hydroxylase activity was also associated with increased keratinocyte expression of TLR2 which further enhances surveillance of infectious bacteria, but also suggests that the effects of vitamin D on TLR expression are likely to be cell-specific. TGFβ and 1,25D may also cooperate to promote expression of other pathways linked to enhanced innate immune responses to infection such as induction of the enzyme 5-lipoxygenase (5-LO) that catalyzes synthesis of leukotrienes. Expression of 5-LO in human monocytes is induced by both 1,25D and TGFβ (Harle et al., 1998), with 1,25D enhancing expression of 5-LO through novel promoter-independent VDRE within exons 10 and 12 and intron M of the 5-LO gene (Stoffers et al., 2010). Although commonly associated with bronchial dilation and asthma, leukotrienes are also known to participate in leukocyte accumulation at sites of infection and phagocytosis of bacteria (Peters-Golden et al., 2005). Leukotrienes have also been shown to trigger the processing of antibacterial CAMP by neutophils (Wan et al., 2007).

Vitamin D-mediated innate immune responses may also be species-specific. VDR-mediated induction of *CAMP* and *DEFB4*, as well as suppression of *HAMP*, appears to be primate-specific; other mammals may therefore utilize alternative innate immunity targets for intracrine 1,25D. For example reactive oxygen species (ROS) can be bacteriocidal; previous studies have shown that macrophages infected with *M. tb* in the presence of 1,25D produce high levels of the superoxide anion ROS via the NADPH oxidase system (Sly et al., 2001). More recent studies have shown that another ROS, nitric oxide (NO), is produced by mouse macrophages as part of innate immune responses to infection, with bacteriocidal consequences (Kohchi et al., 2009). The NO pathway appears to play a pivotal role in mouse responses to *M. tb* infection (Chan et al., 1992), but its importance to human *M. tb* infection is less clear. Moreover, one study using 1,25D and mouse macrophages has reported decreased expression of the enzyme inducible nitric oxide synthase (iNOS) and its NO product, suggesting that the link between vitamin D and NO in innate immune function is more complex than originally thought (Chang et al., 2004). Irrespective of the antibacterial mechanism that is utilized by animals such as mice, it is generally assumed that vitamin D-mediated induction of these responses will occur via the same intracrine monocyte mechanism that has been described for humans. Although expression of CYP27B1 and 1α-hydroxylase activity has been described for murine macrophages *in vitro* (Esteban et al., 2004; Stoffels et al., 2007), the relative importance of this *in vivo* is still unclear. Indeed recent studies using the CYP27B1 KO mouse have suggested that CD8^+^ cytotoxic T cells are the predominant source of extra-renal 1,25D within the murine immune system (Ooi et al., 2014). Further studies are required to fully clarify the physiological importance of this observation.

Genome-wide analyses and associated *ex vivo* and *in vitro* experiments have clearly demonstrated the potential importance of vitamin D in maintaining optimal innate antibacterial responses in humans. However, these studies have also prompted three further crucially important questions: (1) how important is vitamin D for the adaptive immune system? (2) can vitamin D supplementation *in vivo* enhance these antibacterial responses? (3) what happens to the vitamin D system in human immune diseases? Each of these questions will be considered in the remaining sections of this review.

## Vitamin D and antigen presentation

In the seminal DNA array analysis of monocyte TLR2/1 responses by Liu et al that highlighted induction of *CYP27B1* and *VDR* by *M. tb*, it was notable that DCs did not produce the same response when challenged with 19 kDa lipoprotein, despite expressing TLR2/1 (Liu et al., 2006). Monocytes/macrophages belong to the same hematopoietic lineage as DCs, and both types of cells are able to act as antigen-presenting cells (APCs) to promote T cell and B cells responses. Furthermore like, monocytes, DCs express *VDR* and *CYP27B1*, and exhibit an active intracrine vitamin D system (Brennan et al., 1987; Fritsche et al., 2003; Hewison et al., 2003). However, in contrast to monocytes/macrophages, the primary function on intracrine vitamin D in DCs appears to be as a regulator of cell maturation, and ability of DCs to present antigen to T cells (Hewison et al., 2003). Differentiation of DCs toward a mature APC is associated with increased expression of *CYP27B1* but, paradoxically, a reciprocal decrease in *VDR* (Hewison et al., 2003). It therefore seems likely that DCs will utilize a paracrine vitamin D system, with immature DCs expressing VDR and responding to 1,25D produced by mature DCs with lower VDR expression. Such a mechanism may be biologically advantageous in that it allows some DCs to mature and promote T cell activation as part of normal adaptive immune responses, whilst preventing an over-elaboration of this response that could lead to inflammatory complications. A similar pattern of differential regulation of CYP27B1 and VDR has also been described for monocytes differentiating toward macrophages (Kreutz et al., 1993). The importance of 1,25D as a modulator of DC function is endorsed by studies of *VDR* and *CYP27B1* knockout mice, which present with lymphatic abnormalities consistent with increased numbers of mature DCs (Griffin et al., 2001; Panda et al., 2001) and dysregulated DC trafficking (Enioutina et al., 2009).

In a similar fashion to macrophages, DCs can be divided into distinct sub-types, specifically myeloid DCs (mDCs) and plasmacytoid DCs (pDCs). These cells exhibit different cytokine and chemokine profiles and exert complementary effects on T cells; mDCs are efficient promoters of naïve T cell function (Liu, 2005), whilst pDCs are more closely associated with attenuation of T cell function (Steinman et al., 2003). *In vitro*, 1,25D preferentially regulates mDCs, with associated suppression of naïve T cell activation (Penna et al., 2007). However mDC and pDC express similar levels of VDR, so tolerogenic pDC may also respond to 1,25D, possibly via local, intracrine mechanisms (Penna et al., 2007). Alternatively, 1,25D generated by pDCs may not act to regulate pDC maturation but may, instead, act in a paracrine fashion on VDR-expressing T-cells. The ability of vitamin D to influence the differentiation and function of DCs provides another layer of innate immune function that complements its antibacterial properties. However, this interaction between 1,25D and DC will also have downstream effects on cells that interact with APCs, namely cells from the adaptive immune system.

Consistent with the DNA array analyses that shed light on the antibacterial function of vitamin D in monocytes and macrophages (Liu et al., 2006), genome-wide analysis of DCs has revealed diverse responses to vitamin D in these cells. Proteomic analyses using matrix-assisted laser desorption/ionization (MALDI)-time of flight (TOF)/TOF strategies has defined the key proteins associated with tolerogenic responses to 1,25D (Ferreira et al., 2012). Intriguingly, the dominant effect of 1,25D treatment of monocyte-derived DCs described in this study was the alteration of proteins associated with the cytoskeleton and metabolic function. The induction of cytoskeletal proteins was shown to be consistent with DC responses to other tolerogenic steroid hormones, such as glucococorticoids (Ferreira et al., 2012). However, by contrast, the potent effects of 1,25D on metabolic pathways in DCs appear to be distinct from the effects of glucocorticoids. In particular, 1,25D induced significantly more proteins associated with carbohydrate metabolism, gluconeogenesis and the TCA cycle relative to the glucocorticoid dexamethasone, whilst 1,25D and dexamethasone shared induction of other groups of proteins such as those associated with glycolysis (Ferreira et al., 2012). This particular study also illustrates a key advantage of genome/proteome-wide analyses, which is the ability to group changes in gene/protein expression according to specific properties such as metabolism or cytoskeletal function. Moreover, more recent developments allow researchers to utilize tools such as Ingenuity Pathway Analysis (IPA) or DAVID to cluster altered genes and show protein or gene interaction networks (Hong et al., 2009). These “interactomes” provide a picture of the cooperativity of responses to a particular cell treatment. For example, recent proteomic analysis of responses to the synthetic 1,25D analog TX527 in immature and mature DCs showed that 65–75% of the proteins identified as TX527 responsive made up a statistically significant interactome, with some commonality between the two DC types (Ferreira et al., 2009). In this particular study the authors used multiple sets of data including the Biomolecular Interaction Network Database (BIND) (Bader et al., 2003), and the Molecular Interaction Database (MINT) (Zanzoni et al., 2002) to maximize potential interactions. The increasing availability of gene/protein expression databases for different cell types means that this type of strategy is likely to become a more prominent feature of genome-wide expression analyses in the future.

## Vitamin D and adaptive immunity

As outlined above, one of the initial observations linking vitamin D with the immune system was the presence of VDR in activated lymphocytes (Bhalla et al., 1983; Provvedini et al., 1983). The development of lymphocytes takes place in the thymus with VDR being expressed in medullary thymocytes but not in the less mature cortical thymocytes (Ravid et al., 1984). However, once cells leave the thymus and enter the circulation as T or B cells VDR expression is lost until these cells are activated to proliferate by mitogens (Bhalla et al., 1983; Provvedini et al., 1983). Indeed, 1,25D is a potent inhibitor of T-cell proliferation, blocking the transition from early G1 phase to late G1 phase (Bhalla et al., 1984; Nunn et al., 1986), but having no effect on transition from Go (resting) to early G1 or from late G1 to S phase (Rigby et al., 1985). Studies using T cells isolated from lymphatic tissue have shown that expression of VDR and responsiveness to 1,25D is proportional to the rate of cell proliferation (Karmali et al., 1991). Although these early studies have highlighted a role for 1,25D as a regulator of T and B cell proliferation, it has become increasingly clear that the predominant effects of vitamin D on adaptive immune function involve the modulation of T cell phenotype.

T cells consists of several sub-groups including cytotoxic CD8^+^ T cells, natural killer cells, γδ T cells, memory cells, CD4^+^ helper T cells (Th cells), and regulatory T cells (Treg). The best characterized vitamin D responses have been described for Th cells, with 1,25D regulating T cell proliferation and cytokine production (Lemire et al., 1985). Activation of naïve Th cells by antigen and APCs generates pluripotent Th_0_ cells which can then differentiate into further Th sub-groups based on distinct cytokine profiles. Two of these sub-groups, Th_1_ (IL-2, IFNγ, tumor necrosis factor alpha) and Th_2_ (IL-3, IL-4, IL-5, IL-10) T cells, respectively support cell-mediated and humoral immunity (Abbas et al., 1996; Romagnani, 2006). *In vitro* 1,25D inhibits expression of Th_1_ cytokines (Lemire et al., 1995), whilst promoting Th_2_ cytokines (Boonstra et al., 2001). More recently, other Th cell sub-groups have been identified, including interleukin-17 (IL-17)-secreting T-cells (Th_17_ cells) and these cells are also targets for vitamin D. In the autoimmune disease-susceptible non-obese diabetic (NOD) mouse treatment with 1,25D decreased expression of IL-17 (Penna et al., 2006). In a similar fashion, 1,25D suppression of murine retinal autoimmunity involves inhibition of Th17 activity (Tang et al., 2009).

In addition to its effects on Th cells, vitamin D may also act on CD8^+^ cytotoxic T cells which express relatively high levels of VDR (Rigby et al., 1987; Provvedini and Manolagas, 1989; Veldman et al., 2000). As outlined above, CD8^+^ cells in mice have also been reported to express the vitamin D-activating enzyme 1α-hydroxylase (Ooi et al., 2014). CD8^+^ T cells are known to be involved in autoimmune disease such as multiple sclerosis (MS) (Babbe et al., 2000), but do not mediate the effects of 1,25D in suppressing the murine form of MS, experimental autoimmune encepholmyelitis (EAE) (Meehan and DeLuca, 2002). More recent studies have reported a link between vitamin D and a variant of CD8^+^ T cells, CD8αα cells. Unlike CD8^+^ T cells, CD8αα cells are not cytotoxic and may play a role in suppressing gastrointestinal inflammation (Cheroutre and Lambolez, 2008). VDR knockout mice exhibit decreased numbers of CD8αα cells (Yu et al., 2008), due to decreased T cell expression of the chemokine receptor CCR9 preventing T cell homing to the gastrointestinal tract. T cell homing defects provides a potential explanation for the increased colonic inflammation observed in VDR knockout mice when crossed with colitis disease-susceptible mice (Froicu et al., 2003). Vitamin D metabolites may also influence T cell homing in other tissues. In the skin, 1,25D stimulates expression of the chemokine receptor 10 (CCR10) which recognizes the chemokine CCL27 secreted by keratinocytes (Sigmundsdottir et al., 2007).

As well as acting as a modulator of Th cell phenotype and function, vitamin D can also influence adaptive immunity by promoting suppressor T cells known as regulatory T cells (Treg) (Barrat et al., 2002). The precise mechanism by which vitamin D regulates Tregs is still somewhat controversial. Initial studies *in vitro* suggested that the ability of 1,25D to promote CD4^+^ CD25^+^ Treg was due to indirect effects on antigen-presenting DCs, specifically suppression of DC maturation and increased expression of DC cytokines such as CCL22 (Penna et al., 2007). However, subsequent studies have also described direct effects of 1,25D on T cells to generate CTLA4-positive Treg (Jeffery et al., 2009). Significantly, these studies were focused on the use of active 1,25D as the immunomodulator, and it is only in more recent studies that the role of pro-hormone 25D in Treg development has been investigated (Jeffery et al., 2012). Data from this study demonstrated the ability of 25D to promote the generation of Treg through intracrine/paracrine effects on CYP27B1/VDR-expressing DCs. Notably, this report also highlighted the impact of vitamin D binding protein (DBP) on DC responses to 25D, and concluded that non-DBP-bound (free) 25D is the form of 25D that is biologically active for generation of Tregs (Jeffery et al., 2012). The importance of Treg as a facet of vitamin D immunomodulation is illustrated by various studies *in vivo*. In patients with CKD, systemic administration of 1,25D has been shown to increase numbers of circulating Treg (Ardalan et al., 2007). Conversely, in patients with MS, serum concentrations of 25D correlate with Treg activity (Royal et al., 2009; Smolders et al., 2009), underlining the importance of intracrine pathways in mediating effects of vitamin D on adaptive, as well as innate immunity. In mice, topical application of 1,25D (Gorman et al., 2007) or its synthetic analog calcipotriol (Ghoreishi et al., 2009) have been shown to increase numbers of Treg.

The effects of vitamin D on adaptive immunity have to date been very much focused on its ability to modulate T cell proliferation and phenotype. Nevertheless, early studies reported that 1,25D could also suppress the development of immunoglobulin (Ig)-secreting B cells following mitogenic stimulation (Shiozawa et al., 1985; Iho et al., 1986). Initial experiments suggested that the most likely mechanism for this was an indirect effect through inhibition of Th cells (Lemire et al., 1985), but more recent work has shown that 1,25D can suppress the differentiation of two types of B cell, plasma cells and class-switched memory cells, through apparent direct effects (Chen et al., 2007). Other reports have shown that 1,25D can regulate B cell IL-10 (Heine et al., 2008) and CCR10 (Shirakawa et al., 2008), suggesting that the effects of 1,25D on these cells is not restricted to their capacity to produce immunoglobulin.

Although genome-wide screening has played a pivotal role in identifying pivotal mechanisms for the interaction between vitamin D and innate immunity, the same cannot be said for vitamin D and adaptive immunity, where genome-wide analyses have complemented an already well-established field of research. Nevertheless, it is interesting to note reports where this strategy has been applied. In some cases these analyses have revealed a role for the vitamin D system, similar to the seminal studies of *M. tb* induction of CYP27B1 and VDR. For example, transcriptional profiling of γδ T cells reported induction of VDR following activation of these cells with non-peptidic monoalkyl phosphate ligands (Chen et al., 2005). This small sub-set of T cells plays an important role in inflammatory diseases, and it was therefore speculated that 1,25D may act to suppress these cells as part of a more generalized anti-inflammatory response. Further array analyses have also identified VDR as one of a discrete number of genes involved in the formation of B cell germinal centers (Nakayama et al., 2006).

Array analyses have also been used to characterize the gene regulatory effects associated with immunomodulatory responses to 1,25D. These studies have focused primarily on the effects of 1,25D and its synthetic analogs on DCs, with results underlining the ability of 1,25D to promote decreased antigen presentation and a tolerogenic phenotype in these cells (Griffin et al., 2004; Shen and Zheng, 2004; Pedersen et al., 2009; Szeles et al., 2009). Notably, one of these array studies showed that the effects of 1,25D on DC gene expression were independent of DC differentiation status, suggesting a specific role for 1,25D as a regulator of DC function (Szeles et al., 2009). This particular study also reported that key changes in DC gene expression could be achieved using either 1,25D or 25D, further emphasizing the functional importance of the intracrine vitamin D system in these cells. DNA microarray analyses have also been used to assess 1,25D-mediated regulation of gene expression in CD4^+^ Th cells following activation of these cells by phorbol myristate acetate and a calcium ionophore to induce VDR (Mahon et al., 2003). The diverse array of gene targets regulated by 1,25D in this particular array analysis suggests that 1,25D can influence Th cells both directly, as well as via effects on antigen-presenting DCs.

## Vitamin D status and immune function

It is important to recognize that most of the genome-wide analyses that have explored the immunomodulatory effects of vitamin D *in vitro* have focused on treatments using active 1,25D or one of its synthetic analogs. However, as outlined above, pathogen-induction of an intracrine system in cells such as monocytes/macrophages strongly suggests that regulation of immunity *in vivo* is independent of endocrine, systemic 1,25D. Instead it is likely to be primarily driven by local activation of 25D, the major circulating form of vitamin D and determinant of vitamin D status in any given individual. Thus, it is not surprising that translational studies have focused on the relationship between serum 25D and human immune function, including effects on both innate and adaptive immunity.

Epidemiology has shown that vitamin D-insufficiency (serum 25D <30 ng/ml) is associated with increased risk of TB (Wilkinson et al., 2000; Ustianowski et al., 2005; Williams et al., 2008; Wejse et al., 2009). Several clinical trials of vitamin supplementation, as an adjunct to conventional antibiotic therapy, have also been reported with varying success (Nursyam et al., 2006; Martineau et al., 2007; Wejse et al., 2009). Supplementation using 4 × 100,000 IU vitamin D was successful in raising serum concentrations of 25D in TB patients, but this resulted in no overall improvement in sputum conversion time between vitamin D- and placebo-treated patients (Martineau et al., 2011). However, improved sputum conversion time was observed in a specific subset of TB patients with a *Taq1* single nucleotide polymorphism (SNP) within the VDR gene (Martineau et al., 2011), suggesting that genetic factors may influence immune responses to vitamin D supplementation. In a follow-up report to this TB vitamin D supplementation trial, it was shown that raised serum 25D was associated with improved resolution of TB disease (Coussens et al., 2012). Thus, in situations where infectious disease has already become established, it is possible that the role of 25D is primarily focused on anti-inflammatory adaptive immune responses. The link between vitamin D and infection is not restricted to TB. In patients with sepsis, circulating 25D levels have been shown to correlate directly with serum concentrations of CAMP, and inversely with critical illness in these patients (Jeng et al., 2009). Low serum 25D has also been linked to upper respiratory infections such as influenza (Cannell et al., 2006), and in patients with CKD low serum is associated with increased risk of infection and mortality (Gombart et al., 2009a).

**Figure 2:**
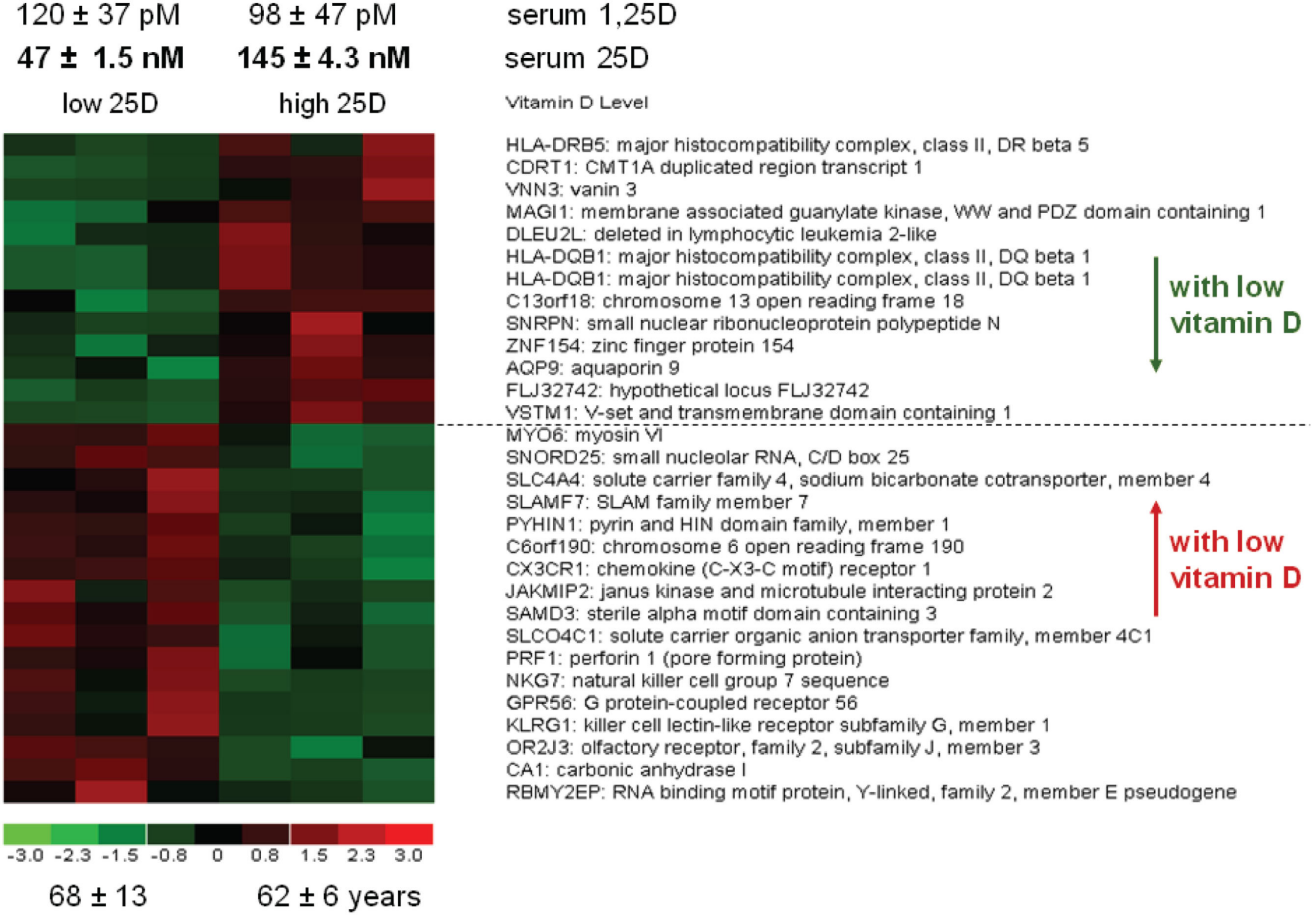
**Altered gene expression in leukocytes from vitamin D-deficient donors.** Peripheral blood mononuclear cells (PBMC) were obtained from 6 healthy donors who had serum 25D levels that were either low (47 nM, 18.8 ng/ml) or high (145 nM, 58 ng/ml). After isolation of RNA and generation of cDNA, gene expression analyses in the PBMC samples was carried out by DNA array analyses (Affymetrix Exon Expr Chip.HuGene-1_0-st-v1). Data are shown as genes that were either increased (red) or decreased (green) in low 25D donors relative to high 25D donors (>1.5-fold change, *p* < 0.05, unpaired *t*-test).

To date, the application of genome-wide analyses to further elucidate the impact of serum vitamin D (25D) status on immune function has been limited. In a recent study by Holick and colleagues, array analysis of gene expression in peripheral blood mononuclear cells from vitamin D-sufficient (serum 25D >20 ng/ml, *n* = 4 subjects) and vitamin D-deficient (serum 25D <20 ng/ml, *n* = 4 subjects) revealed 66 differentially expressed genes (>1.5-fold change, *p* < 0.01) (Hossein-nezhad et al., 2013). However, after vitamin D supplementation (2000 IU vitamin D/day for 2 months), there was no significant change in the expression of these genes, even though serum 25D levels were increased in both sufficient and deficient subjects (Hossein-nezhad et al., 2013). Nevertheless, 291 additional genes were found to be differentially expressed in peripheral blood mononuclear cells following vitamin D supplementation (>1.5-fold, *p* < 0.01) (Hossein-nezhad et al., 2013). Similar array analyses carried out by our group using peripheral blood mononuclear cells from elderly vitamin D-deficient (18.8 ± 0.6 ng/ml serum 25D) and vitamin D–sufficient (58 ± 1.7 ng/ml serum 25D) patients, revealed 30 differentially regulated genes (Figure 2). These variations in gene expression occurred against a backdrop of no difference in serum 1,25D concentrations between vitamin D-sufficient and -deficient groups, underlining the importance of 25D, and the intracrine vitamin D system as regulators of immune cell function. In both this study and the Holick report, array analyses were carried out using mixed populations of systemic immune cells including both innate immunity APCs and lymphocytes of the adaptive immune system. The array analyses will therefore encapsulate both intracrine and paracrine activity of 25D, but will also reflect inherent donor to donor variations in immune cell composition.

Genome-wide analyses of immune responses to altered vitamin D status in mice are also very limited. Data from our group using colon tissue from vitamin D-deficient (serum 25D = 2.5 ± 0.1 ng/ml) vs. vitamin D-sufficient (serum 25D = 24.4 ± 1.8 ng/ml) identified 31 genes that were differentially expressed >2-fold (*p* < 0.01) (Lagishetty et al., 2010). Amongst these, vitamin D-deficient mice showed decreased expression of angiogenin-4 (Ang4), an antimicrobial protein which acts to minimize tissue invasion by enteric bacteria (Hooper et al., 2003). Further studies showed that decreased Ang4 in vitamin D-deficient mice was associated with increased levels of bacteria in the colon epithelium, consistent with compromised innate immune surveillance. Given that dysregulation of innate immune responses to enteric bacteria has been linked to the initiation of tissue inflammation associated with some types of inflammatory bowel disease (Packey and Sartor, 2009), it is possible that vitamin D plays a role in protecting against this disease via the induction of antibacterial Ang4.

Another genome-wide strategy with implications for vitamin D and the immune system, arose from studies aimed at determining the genetic component of vitamin D-deficiency. A recent Genome-Wide Association Study of almost 34,000 individuals showed that SNPs within the *DBP* gene are a key inherited determinant of low vitamin D status (serum 25D <75 nM or 30 ng/ml). Gene variations in *DBP* appear to act by influencing the serum concentrations of DBP protein (Lauridsen et al., 2001) which are known to be linked to serum levels of total 25D and 1,25D (Lauridsen et al., 2005; Wang et al., 2010a). Studies of other *DBP* SNPs suggest that genetic variants of *DBP* are linked to different binding affinities of 25D for DBP protein (Arnaud and Constans, 1993). Both the concentration and binding affinity of DBP protein are important for the serum transport of vitamin D metabolites (notably 25D which binds to DBP with a higher affinity than 1,25D). However, DBP concentration and affinity also define the amount of 25D that is *not* bound to DBP. This “free” or “bioavailable” fraction of circulating 25D appears to be the form that accesses target cells such as monocytes (Chun et al., 2012), presumably via passive diffusion of lipid-soluble 25D through cell membranes—the so-called “free hormone hypothesis.” Studies by our group have shown that antibacterial responses to 25D *in vitro* are more pronounced with low affinity forms of DBP that support higher levels of free 25D (Chun et al., 2010). Studies to date have been based on mathematical estimations of free 25D from total serum concentrations of 25D and DBP (Chun et al., 2012). However, future strategies using actual physical measurement of free 25D will greatly help to clarify the precise importance of total vs. free 25D in determining immune responses to vitamin D.

## Immune disease and the dysregulation of vitamin D

Genome-wide strategies have played a pivotal role in elucidating the core mechanisms that trigger the intracrine vitamin D system and associated immune responses in cells such as monocytes/macrophages and DCs. Whether these studies have been carried out using freshly isolated preparations of immune cells, or cultured immune cells the resulting data have reflected the potential vitamin D-mediated responses that may occur following a pathogen challenge. What is less clear is how these responses function under conditions of actual human immune disease. An illuminating example of this strategy is provided by the disease leprosy which, like TB, involves a mycobacterial infection—in this case *Mycobacterium leprae* (*M. lep*) or *Mycobacterium lepromatis. S*imilar to TB, vitamin D was at one time considered to be a putative therapy for leprosy (Herrera, 1949). However, unlike TB, leprosy can be divided into different disease sub-types, notably tuberculoid leprosy (T-lep) and lepromatous leprosy (L-lep). These two forms of leprosy have very different immune profiles and prognoses (Britton and Lockwood, 2004). DNA array analyses to define the gene expression profiles associated with the T-lep and L-lep forms of leprosy, highlighted elevated expression of CYP27B1, CYP24A1, and VDR in T-lep vs. L-lep lesions (Montoya et al., 2009). The over-arching conclusion from these studies is that the less aggressive form of leprosy, T-lep, is manifested by an intact vitamin D intracrine system that is able to support antibacterial responses to vitamin D. By contrast, L-lep, which is characterized by a high level of macrophage *M. lep* infection, and has a poor prognosis, exhibits an impaired vitamin D intracrine system. Thus, for patients with L-lep, successful elevation of serum 25D concentrations may be less effective in promoting intracrine-mediated regulation of antibacterial responses.

**Figure 3:**
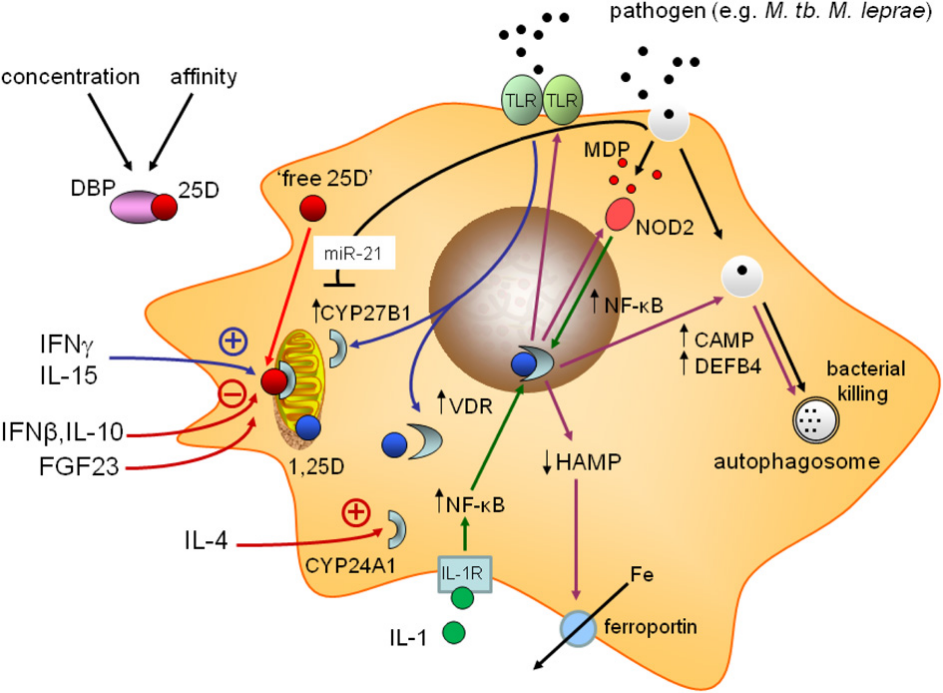
**Mechanisms for corruption of vitamin D-mediated antibacterial responses in monocytes.** Schematic representation of monocyte/macrophage responses to infection with a pathogen such as *Mycobacterium tuberculosis* (*M. tb*), and associated adjunct signals. Positive effects on the vitamin D intracrine system include: induction of CYP27B1 expression by cytokines such as interferon γ (IFNγ) and interleukin-15 (IL-15). Negative effects include suppression of CYP27B1 by IL-10 and IFNβ, and induction of 24-hydroxylase activity by IL-4. Fibroblast growth factor 23 (FGF23) also suppresses CYP27B1 and microRNA-21 (miR-21) associated with some pathogen infections (e.g., *M. leprae*) can suppress expression of CYP27B1 by degradation of RNA and/or translation. The serum vitamin D binding protein (DBP) may also attenuate intracrine vitamin D by restricting monocyte bioavailability of free 25D.

Several questions have arisen from the studies of vitamin D and leprosy. The first concerns the mechanism by which the vitamin D intracrine system is corrupted in L-lep patients. One possibility is that the T cell cytokine profiles that are characteristic of L-lep (e.g., increased IL-4, IL-10, and IFNα/β) exert a detrimental adjunct effect on the underlying TLR2/1-induced intracrine vitamin D system. At the same time, cytokine profiles associated with T-lep (e.g., increased IFNγ) may have more beneficial adjunct effects. Subsequent experiments *in vitro* support this hypothesis, with the Th1 cytokine IFNγ enhancing TLR2/1-induced vitamin D-activation and associated antibacterial activity (Edfeldt et al., 2010; Fabri et al., 2011). Conversely, the L-lep cytokines IL-4 (Edfeldt et al., 2010), IFNβ (Teles et al., 2013), and IL-10 (Teles et al., 2013) suppress antibacterial production (Figure 3). The effect of IFNβ appears to be mediated via IL-10 which acts to suppress expression of CYP27B1 (Teles et al., 2013), whereas IL-4 appears to act by stimulating activity of the vitamin D catabolic enzyme CYP24A1 (Edfeldt et al., 2010). The collective conclusion from these studies is that specific human diseases are characterized by T cell cytokines that act to either promote or corrupt the underlying pathogen-PRR-driven vitamin D intracrine system. Cytokine profiles such as this have also been described for active and inactive TB (Berry et al., 2010; Maertzdorf et al., 2012), providing an additional perspective on the varying success of vitamin D supplementation trials with this disease. Disease itself may therefore play a fundamental role in determining the efficacy of immunomodulatory vitamin D for any given patient; for example, it is possible that for diseases such as L-lep, higher levels of serum 25D will be required to achieve a specific antibacterial response. This is clearly an important topic for future research.

The differential regulation of the intracrine vitamin D pathway in T-lep and L-lep has also provided a platform for genome-wide analyses aimed at identifying factors other than T cell cytokines that may be involved in corrupting monocyte vitamin D responses. In a follow-up to the previous DNA array studies for leprosy, further array analysis of T-lep and L-lep tissues revealed distinct patterns of microRNA (miRNA) expression in these tissues (Liu et al., 2012). Recent studies have shown that miRNAs play a key role in fine-tuning gene expression by interacting with RNA to silence gene expression either by degrading transcripts, or by blocking their translation (Ketting, 2011). In L-lep, 16 miRNAs were found to be differentially induced relative to T-lep tissue, with miRNA-21 (miR-21) being the most prominent of these (Liu et al., 2012). In the context of innate immunity and leprosy, miR-21 may target several important mechanisms, including suppression of IL-1 expression which may, in turn, attenuate intracrine induction of antibacterial DEFB4 (see Figure 3). However, importantly, miR-21 is also predicted to interact with *CYP27B1* mRNA and suppress activity of 1α-hydroxylase and decrease localized synthesis of 1,25D. *In vitro*, siRNA knockdown of miR-21 in *M. lep* infected monocytes, restored CYP27B1 expression and 25D-mediated antibacterial responses (Liu et al., 2012). Despite these observations, relatively little is known about how miRNAs corrupt vitamin D signaling in disease situations. Based on genome-wide *in silico* analysis of miRNA target sequences, multiple miRNAs are predicted to influence the expression of proteins associated with vitamin D metabolism and signaling (reviewed in Lisse et al., 2013a). However, other than studies of miR-21, there have been few studies to validate the predicted effects of miRNAs on the vitamin D system. Analysis of ovarian granulosa and breast cancer cells has demonstrated increased expression of miR-125B in these tissue, and a concomitant dysregulation of two of its targets, *VDR* and *CYP24A1* mRNAs (Mohri et al., 2009). It seems likely that future studies will identify other miRNAs that modulate the vitamin D intracrine system in immune cells under disease conditions. Moreover, it is important to recognize that vitamin D itself is a potent regulator of miRNAs. To date, these studies have focused on cancer (Wang et al., 2009, 2011), and bone cells (Lisse et al., 2013b), but similar future studies of 1,25D-regulated non-coding RNAs in immune cells may provide an entirely new perspective on the immunomodulatory actions of vitamin D.

Some important questions about human disease and the immunomodulatory effects of vitamin D remain unanswered. For example, it is still not clear why there is aberrant synthesis of 1,25D by macrophages in granulomatous diseases (Kallas et al., 2010). It is also unclear what effect, if any, viral pathogens such as hepatitis C or HIV have on innate and adaptive immune actions of vitamin D, although HIV infection of some cells has been shown to suppress expression of *VDR* (Chandel et al., 2013). Future studies of vitamin D and the immune system may also explore non-traditional targets for immune regulation. For example, patients with end-stage kidney disease who routinely use dialysis are at high risk of infection and associated mortalities. These patients are also commonly vitamin D-deficient (Zehnder et al., 2007), and this may impair normal innate immune responses to infection. However, as with TB and leprosy, additional disease factors may act to further compromise the intracrine vitamin D system in these patients. Notably, circulating levels of fibroblast growth factor 23 (FGF23), which plays a key role in the endocrine regulation of phosphate homeostasis, are elevated very early in kidney disease (Danziger, 2008; Isakova et al., 2011). One of the important actions of FGF23 is to suppress renal production of 1,25D through the suppression of CYP27B1 expression (Shimada et al., 2004); in this way FGF23 acts as a counterpoint to parathyroid hormone which stimulates CYP27B1 and renal 1,25D production. Until recently, the effects of FGF23 were thought to be restricted to the mineral homeostasis endocrine system. However, work by our group has shown that FGF23 can also act on monocytes to suppress expression of CYP27B1 and the intracrine induction of antibacterial proteins (Bacchetta et al., 2013a). These data highlight a mechanism by which renal disease may compromise vitamin D-mediated immune function, similar to that observed for cytokines associated with infectious disease (see Figure 3). As well as providing an explanation for the increased risk of infection in kidney disease patients, these results also suggest a hitherto unrecognized link between the vitamin D endocrine system and its intracrine immune counterpart.

## Perspectives

For many years, the link between vitamin D and the immune system was considered to be a non-classical response with only a pathophysiological relevance. The advent of genome-wide analyses has enabled a complete change in this perspective by providing an unbiased picture of how the vitamin D system is induced by pathogens, and how the resulting intracrine cellular machinery can promote both innate and adaptive immune responses to the pathogen. A key challenge going forward will be to relate these mechanisms to patient vitamin D status, and this is likely to herald a new wave of genome-wide analyses linked to placebo-controlled vitamin D supplementation trials. Interpretation of these studies is likely to be complex. Recent genome-wide analysis of patient tissues has shown that some immune diseases are characterized by corruption of the vitamin D system, so that conventional notions of vitamin D-sufficiency and vitamin D-deficiency may be very different for patients with specific diseases. A key objective for future studies will be to determine whether vitamin D-mediated-immune function is also applicable to mouse models, where genome-wide screening will help to identify immune targets that are related to, or distinct from, human data. Future studies will also need to better characterize disease corruption of vitamin D responses. Screening for disease-specific microRNAs will be particularly important to identify non-coding RNAs that can target components of the intracrine vitamin D. The current shift away from DNA array technology to RNAseq strategies will help to achieve these new objectives in a single genome-wide screen, at increasingly affordable prices.

### Conflict of interest statement

The authors declare that the research was conducted in the absence of any commercial or financial relationships that could be construed as a potential conflict of interest.
